# Disease of the Antrum

**Published:** 1891-09

**Authors:** S. Woolverton

**Affiliations:** London, Ont.


ARTICLE VI.

### DISEASE OF THE ANTRUM.

BY S. WOOLVERTON, L. D. S., LONDON, ONT.

Read before the Ontario Dental Associatioa, July 21.

Alveolar dental abscess is a common surgical affection, attended with great suffering, and more or less serious consequences according to the condition of the patient, the structure of the alveolar tissues concerned, and the location of the tooth.

The relation of the antrum of Highmore to the roots of the teeth in the upper jaw is such, that when disease of these organs occurs, the discharge is liable to enter this cavity, and nearly all diseases which we are called on to treat will be found to come from abscessed teeth, and the removal of the offending tooth, or teeth, will usually be the cure of the trouble.

In examining a human skull, properly divided for this purpose, we find this sinus presents great variations in individual cases. In some cases there is a heavy lamina of bone between the roots of the teeth and the cavity, but occasionally a case is met with in which the roots of the teeth actually project into it, covered, however, with a thin lamina of bone, in addition to the mucous membrane. Is it any wonder, therefore, that serious consequences will often arise from this, especially if the pus is not fully discharged by way of the nostril on the affected side? The pus may also find its way into the cavity, even when there is a considerable thickness of bone between it and the root of the tooth.

The disease may be either acute or chronic. In the acute forms of abscess the general law is, that the burrowing of the pus will go in the direction in which there is the least resistance; on the other hand, the movement in chronic forms is very gradual, and is largely guided by gravitation, and therefore sinks to the lowest point. The rule is, that we find will the point of discharge below the source of the pus, and this is the reason that we find that by far the larger number of alveolar abscesses that discharge on the face are situated on the lower jaw. The burrowing of pus in the chronic forms of abscess form a very important element in their history. This presents the widest variations, and is sometimes the source of much perplexity to the physician or dentist. The diagnosis and treatment of the disease, although plain, are in many instances wholly misunderstood, and too frequently are we called upon to treat chronic cases that might have been cured at a much earlier stage of the disease, and it is a matter of regret, that some medical men (and even dentists) have so little knowledge on this subject; hence the need of specialists in this line.

The treatment of the alveolar abscess, in a vast majority of cases, presents but little difficulty. It consists in a thorough evacuation of the pus from the cavity, and cleaning and disinfecting it, and relates more especially to the removal of the cause perpetuating the discharge of the pus Among the many antiseptics in use, there are none which answer all the requirements better than carbolic acid and peroxide of hydrogen.

The medication should take place through the opening, which will generally be found through one or more of the sockets from which the teeth have been taken, and these may easily be enlarged if necessary, and not through the natural opening from the antrum through the middle meatus of the nose, as I have seen it done, without beneficial results. It is said by some, that we obtain better results by using an atomizer than from the syringe, in applying our remedies, the spray more thoroughly reaching the parts, but in my opinion, a common rubber bulb syringe is superior to either. In the treatment we are apt to do too much than not enough.

Nature is frequently the best physician, and treating intelligently, so as to assist nature, is generally sufficient, and in cases of this kind you will be astonished to see how favorable the symptoms become, and the artificial opening closing with healthy granulations until the whole trouble passes away.

I will now relate the history of two of the most important cases that I have met with. In each instance the first upper molar was the exciting cause of the trouble, and both on the left side of the face. My observation of this disease leads me to think that this trouble is more apt to occur on the left than on the right side of the face. Why this should be so I will leave older heads to determine.

### AN ACUTE CASE OF ABSCESS OF ANTRUM.

About two years ago a laborer in the car-shops of London came to consult me about a discharge that was coming from the inner canthus of the eye, and for which he had been under treatment by a physician for some weeks, who was treating it locally. The discharge was very profuse, and exceedingly offensive, so much so, that I had no desire to treat it. After hearing the history of the case, and establishing a true diagnosis of the disease, by examining the teeth on the affected side, I found that the first superior molar had a dead pulp,and was painful on percussion. I removed this tooth, and found a direct opening into the antrum. I then sent him to his physician with instructions to treat through the opening thus made. I saw him a short time afterwards, and he had made a rapid recovery, but with an ugly scar at the corner of the eye, where the pus had forced its way out. Timely treatment would have prevented this, and saved weeks of suffering as well as expense, as he was not able to attend to his work in the meantime.

CASE, No. 2, *Chronic*.—Miss B., of London, aged 19, suffered from this disease, for which she had been under treatment for more than six years previous, with four different physicians, who failed to bring about a cure, owing to a wrong diagnosis of the case. True diagnosis, as we all know, is the first requisite in the treatment of any disease. She had been treated for nasal catarrh symptoms. Offensive breath, appetite gone, languid and despondent, and had almost given up hope of being cured. Necrosis had also set in, and the spongy bones around the natural opening from the antrum were softened and coming away. This tended to make the treatment much more tedious than it would have been otherwise. When I first saw her there was a profuse discharge of mucous from the nostril on the affected side. The lower eyelid was quite red and swollen, the conjunctive was much inflamed, and there was a sense of heaviness in the left cheek, and other symptoms that accompany this disease. The diagnosis offered no difficulty in this case. The treatment was simple and efficacious. The removal of the first superior molar, which I found in a carious condition, allowed me a free entrance into the antral cavity. The opening in this case allowed me to treat freely, and had no difficulty in keeping it open by means of small pieces of slippery elm bark inserted into the opening. The cavity was kept clean by frequent and copious washings of warm water and salt. The case was under treatment for about six months. During this time I used injections of carbolic acid diluted, carbolated iodine, tinct. opii camph., and last but not least, peroxide of hydrogen.

The patient made a complete recovery, and her general health has been very much improved.

Her last physician told her that a cure could not be effected without her undergoing a surgical operation, and she had almost resolved to go to Toronto to have the operation performed, when she came to me for consultation.—*Dominion Dental Journal.*

